# Formal Verification of Control Modules in Cyber-Physical Systems

**DOI:** 10.3390/s20185154

**Published:** 2020-09-10

**Authors:** Iwona Grobelna

**Affiliations:** Institute of Automatics, Electronics and Electrical Engineering, University of Zielona Góra, 65-417 Zielona Góra, Poland; i.grobelna@iee.uz.zgora.pl

**Keywords:** control systems, cyber-physical systems, formal verification, manufacturing systems, model checking

## Abstract

The paper proposes a novel formal verification method for a state-based control module of a cyber-physical system. The initial specification in the form of user-friendly UML state machine diagrams is written as an abstract rule-based logical model. The logical model is then used both for formal verification using the model checking technique and for prototype implementation in FPGA devices. The model is automatically transformed into a verifiable model in nuXmv format and into synthesizable code in VHDL language, which ensures that the resulting models are consistent with each other. It also allows the early detection of any errors related to the specification. A case study of a manufacturing automation system is presented to illustrate the approach.

## 1. Introduction

Cyber-physical systems are found in every area of our life. They are used in vehicular systems, medical and health-care systems, smart homes and buildings, social networks and gaming, power and thermal management systems, data centers, electric power grids and energy or networking systems [1,2,3,4,5,6,7]. A cyber-physical system (CPS) is an integration of computation with physical processes, the behavior of which is defined by cyber and physical parts of the system [8]. Hence, the design of such systems requires an understanding of the joint dynamics of computers, software, networks and physical processes. A unified 5-level architecture has been proposed as a guideline for implementation of cyber-physical systems (the 5C architecture [9]). What distinguishes the CPS from any other existing system is the connection of the physical world with the cyber system. The amount of information gathered from the environment is enormous and appropriate mechanisms are needed to efficiently analyze it and make appropriate decisions.

The purpose of the control part of a cyber-physical system is to monitor the environment and to control specific objects. A sense of the world is created by use of multiple input signals, coming from various sensors or coexisting systems, e.g., a computer vision system. The influence on the outside world is effected by various output signals, which can control the executive elements or become input elements for other parts of a cyber-physical system.

Control processes are already well described in the literature. There are various specification techniques of control processes [10], including Petri nets (PN) [11,12,13], statecharts [14] and diagrams of the Unified Modelling Language (UML) [15,16]. There are also some approaches to using these specification techniques in the domain of cyber-physical systems, such as [17,18,19,20,21,22,23]. Each specification technique has its advantages and disadvantages. Petri nets, as a mathematical model, has the wide support of analysis and verification methods [12,24,25]. Statecharts [14] were introduced as a state-based representation of a reactive system. UML diagrams in turn, are simpler, more user-friendly and more frequently used in the industry. The use of UML improves the information flow between team members and provides better understanding of system behavior. Moreover, some of the verification techniques are also applicable, including, e.g., simple model checking of activity diagrams [26] or state machine diagrams [27].

When designing any system, and especially a reliable and safe system, it is important to verify the specification before further stages of system development [28]. The earlier any errors are detected, the faster they can be repaired and the cheaper the costs are. Formal methods [29,30] can be applied at the early stage of system development, namely, at the specification stage. Indeed, the specification can be verified against some user-defined requirements, expressed as temporal logic formulas [31], using the model checking technique [32]. This then gives an answer to the question of whether the requirements hold, and if otherwise, appropriate counterexamples are generated to track the undesirable situation.

In the paper a novel formal verification method for a state-based control part of a CPS is proposed. The control part is expressed by means of UML state machine diagrams, and the model checking technique (with the nuXmv tool [33]) is used to validate it. The original abstract rule-based model (described in detail in [12]) is used as an intermediate model between the initial specification and the verification and implementation. The transformations between models ensure that the resulting models are consistent with each other, and ultimately, final prototype implementation can be achieved that reflects the already verified specification. The preliminary outline of the method, not concerning the cyber-physical systems yet and without any formal algorithms, was sketched in a short conference paper [27]. There, a rule-based approach to model checking of UML state machines was briefly introduced as an idea that could possibly be extended in the future. Now we give the details and algorithmic representation of the method and show how it can be used in the design of a control part of a cyber-physical system. An in-depth exhaustive analysis of the state-of-the-art is given, along with a detailed case study to illustrate the approach. Additionally, the proposed method has been experimentally verified; we focused especially on formal verification of the state-based control part of cyber-physical systems.

The main contributions of the paper can be summarized as follows:

▪ A novel rule-based representation of a state-based control part in a cyber-physical system is proposed, which is also a basis for the automatic generation of a synthesizable model in the VHDL language (Very High Speed Integrated Circuit Hardware Description Language) for prototype implementation in an FPGA (Field-Programmable Gate Array) device.
▪ The model checking technique is applied and the abstract rule-based logical model is automatically transformed into a verifiable model (in the nuXmv format).
▪ The proposed method is supported by formal algorithms.
▪ The simplicity of UML diagrams is combined with the benefits of formal methods of verification.
▪ The approach can successfully be used in design of control part of automation manufacturing cyber-physical systems.

Let us shortly justify the choices made. In this paper, UML state machine diagrams are used because of their simplicity, readability and common use in the industry. The model checking technique is applied because of its well-established contribution in formal verification that enables the achievement of reliable systems that fulfil the initial requirements. VHDL language and FPGAs are used on account of the possibilities of FPGA devices, especially partial reconfiguration and very short time between an input and its response. VHDL code can also be simulated before synthesis. The developed *m2vs* tool was used for experiments and for automatic generation of verifiable and synthesizable models based on a rule-based logical model. Finally, the nuXmv model checker was applied as an up-to-date tool used in several industrial projects.

The rest of the paper is structured as follows. Section 2 presents related work, focusing especially on cyber-physical systems, their specification and verification, but also on the verification of UML state machine diagrams. Section 3 introduces the proposed method of formal verification, based on the use of an abstract rule-based logical model. Section 4 illustrates the proposed method with a case study example of a modern automation manufacturing system. Section 5 provides some discussion, and finally, Section 6 summarizes and concludes the paper.

## 2. Related Work

Although at the beginning of the cyber-physical systems era the existing classical approaches could not be directly applied to the CPS domain, in recent years much progress has been observed and various valuable research projects have been conducted. Let us present some of the work done that has contributed to the observable evolution of the state-of-the-art.

### 2.1. Design of CPS

Many survey papers in the literature indicate that the design of cyber-physical systems should be paid much more attention. The report presented in [34] stresses the importance of further research in the CPS domain. New models, algorithms, methods and tools are needed to incorporate verification and validation of software and systems at the control stage of design. Moreover, standardized abstractions for modular design and development of cyber-physical systems should be elaborated. In [35] design challenges for CPS are presented. It is reported that for the full potential of cyber-physical systems, computing and networking abstractions have to be rebuilt, as it is not sufficient solely to improve design processes, raise the level of abstraction or verify designs that are built on the abstractions before the era of cyber-physical systems.

In [36] another short survey of CPS is presented. The research progress from various perspectives is summarized. Although there are several existing model-based development methods, they cannot be applied directly in a CPS. Another survey on concepts, applications and challenges [37] reveals that revolutionary design approaches are necessary to achieve overall system objectives. Future challenges related, e.g., to design, functionality or verification are also identified in [5]. In [38] the potential for CPSs in societally important application domains is outlined, with a comprehensive discussion on design and development from the software engineering point of view. Other articles review recent advancements in design and operation [4], security [39] or formal verification [40] of power grids in the area of cyber-physical systems. The development method for cyber-physical systems based on a model driven approach with Petri nets and quality assurance is proposed in [41]. The CPS for safe human-robot collaboration in a shared workplace, based on real-time evaluation of safety distance, is shown in [42]. A model-integrated development framework with both hardware and software components (along with the interactions between them) specified with the EsMoL modelling language is presented in [43]. Model-driven software development for CPSs is also discussed in [44,45].

### 2.2. Verification of CPS and UML State Machines

Verification and validation of cyber-physical systems are widely studied in the literature. A review of papers along with additional interviews [46] of their authors reveals that existing formal and simulation methods are insufficient for supporting the development of cyber-physical systems for general purpose, and that classical software engineering methods cannot be applied directly. Formal methods need to be made more comprehensive to capture the heterogeneity of CPS, and in addition required user intervention should be reduced. Respondents identified a lack of formal connection to models of physical systems and tended not to deeply consider formal models of physical systems during development, finding the available software models inadequate. Furthermore, model-driven development is said to be far from mature in the CPS domain. Formal verification of BNDC (bisimulation-based non deducibility on compositions) properties in CPS with a stochastic process algebra (SPA) and model checking technique is proposed in [47]. Statistical model checking, valuable for systems with large state spaces, is introduced in [48]. Online building and verification of models describing time-bounded behavior of cyber-physical systems is presented in [49]. The abstraction methodology of hardware, software and environment models of the CPS is shown in [50], with labelled hybrid Petri nets as a formalism. Domain-specific model checking is proposed in [51], with MechatronicUML, UPPAAL model checker and mechanisms for translation and back-translation between them.

A number of research papers are also focused on the security of CPS systems. A brief survey of CPS security identifying key research challenges for the future is given in [52]. In [53] a survey of CPS security is given, differing from other surveys in the fact that three perspectives are taken into account, namely, a security perspective (threats, attacks, etc.), a CPS component perspective and a CPS perspective. Challenges for the CPS security are also identified. In [54] the state-of-the-art of cyber-physical system security from an automatic control perspective is presented. Security is one of the main scientific challenges for CPS, especially considering uncertainty in the environment, security attacks and errors in physical devices. A systematic study of 138 research papers shows that security is a relatively young research domain with a lot of perspectives for research in the forthcoming years.

Some uncertain environmental situations with the likelihood of occurrence can be identified during the formal verification process; hence, the model checking method based on the rule-based logical model and related to CPS control part perspective, proposed in this article, can contribute to at least a partial increase in security.

Verification of contemporary CPS can be done with the use of theorem proving. Existing theorem provers have to consider various aspects of CPS. The survey on developed tools is provided in [55] with the conclusion that only one dedicated theorem prover (KeYMaera) has been developed for analyzing hybrid systems. Theorem proving is also used in [56], with a conceptual framework for the development of complex systems based on higher-order logic specification. Tight integration of verification with model-driven development and simulation makes it possible to address both digital and analog safety-critical systems. A top-down design methodology of cyber-physical models with integrated validation and formal verification is proposed in [57,58] (with the model checking technique). A given initial abstract model is formally verified to reveal any property violations, then a mapping to a concrete model is determined, with validation of whether it is realistic or not. However, the two models are not exactly the same, with the abstract model being an over-approximation of the concrete model, which enforces multiple checking. A verification process involving the application of model checking, runtime verification and analysis of software behavior is presented in [59]. Timing properties are checked and the scheduling problem in a CPS design is evaluated. It is concluded that the use of a single verification technique might not be enough to cover the totality of satisfaction in properties, especially in complex CPSs.

The concept of an end-to-end methodology helping to identify specifications from various sources, automatically creating formal specifications and applying them to the verification of cyber-physical systems is discussed in [60]. Digital twin technology is used to help accelerate the development and start verification earlier using multi-models combined with hardware-in-the-loop and software-in-the-loop co-simulations. Model checking of specialized cyber-physical energy systems and complex power electronics systems is proposed in [61,62], with the use of a modelling and simulation-based software. A practical verification framework filling the gaps between model-based development and the formal verification process is proposed in [63], and consists of a model transformation method for the plant models of CPSs, including differential algebraic equations to equivalent models without them, in order to reduce verification complexity. The model simplification method automatically simplifies bond-graph models by replacing complex components with simpler components for further verification of overhead reductions. The proposed verification framework has been successfully applied to safety verification of an automotive brake control system. Another framework for CPS modelling and verification based on dynamic logic is proposed in [64], with HybridUML to model CPS and a method based on model transformation mapping from HybridUML to Hybrid Program.

A survey on automated symbolic verification and its application for synthesizing cyber-physical systems is provided in [65], with a discussion on recent advances in symbolic model checking techniques and their applications to control synthesis. Stringent constraints imposed by underlying hardware along with system behavior models must be considered during verification and synthesis. Indeed, formal synthesis consists of designing correct systems (according to evaluation procedures), and thus, formal synthesis is dependent on formal verification and both are based on sound models of systems and properties.

There exist also some approaches to formal verification of classical UML state machine diagrams, regarding especially UML in version 1.x. However, the existing approaches do not address logic controller design and do not support additional synthesis opportunities. In [66] state machines are used in the software engineering domain. Protocol models in UML version 1.4 are considered in [67]. In [68] all executions of a UML system are encoded directly into a Boolean propositional formula and no existing model checker tool is used. In [69] abstraction and refinement are applied in the verification of behavioral UML models in order to make the model checking easier. The direct transformation method of UML state machines into SMV specification is proposed in [70], with a tool proposition for integration of formal methods allowing joint verification of activity and state diagrams. In [71] state machines are transformed into colored Petri nets for verification purposes. In [72] UML state machines for embedded software design are executed and formally verified with the UMerL tool by means of a translation of behavioral information into Erlang.

An extensive analysis of the literature showed that formal verification of cyber-physical systems is a current and indispensable research topic. It allows not only the improvement of the quality and reliability of a cyber-physical system, but can also contribute to an increase in security, especially when taking into account possible attacks and uncertainty in the environment.

The following section introduces a novel approach to formal verification of a state-based control part of a cyber-physical system, using the model checking technique and the abstract rule-based logical model.

## 3. The Proposed Rule-Based Approach

In this work the control part of a cyber-physical system is specified as a state machine diagram of the UML language (in version 2.x). UML state machines as behavioral diagrams of the Unified Modelling Language allow the modelling of discrete system behavior. They depict various states that a system may be in, together with transitions between these states. Their basic elements include states, transitions as progressions from one state to another, an initial pseudo-state and a final state. Furthermore, it is possible to group some states into a composite state or to use orthogonal states to model the concurrency. State machines in logic controller design additionally take into account the presence of input and output signals. Inside a state some information about output signal activity can be provided through the labels for identification of ongoing behavior, performed as long as the element is in the state (the so-called do activity). In contrast, input signals may be treated as guards or triggers for transitions.

### 3.1. Preliminaries

The abstract rule-based logical model is a formal notation of a control process with a predefined syntax that can be automatically transformed into a verifiable model and a synthesizable model. It describes desired system behavior and formally can be defined as follows [24]:

*A rule-based logical model is a formal notation of control system behavior, consisting of the five following sections: definition of variables (here: states, input and output signals) Var = {S, X, Y}; initial values of variables Var_0_ = {S_0_, X_0_, Y_0_}; rules as descriptions of transitions T; input signal changes and output signal changes.*

The elements of the UML machine diagram are directly reflected in the rule-based logical model. Transitions between states build the rules—with some pre- and post-conditions (separated with the temporal logic operator *X*). States may have assigned some output signals (as do activities), while transitions may have assigned some input signals as the guards. Input signal changes listed in the rule-based logical model are then used only for generation of a verifiable model to eliminate the state explosion problem (so the changes occur only before related transitions).

### 3.2. General Description

**Figure 1:**
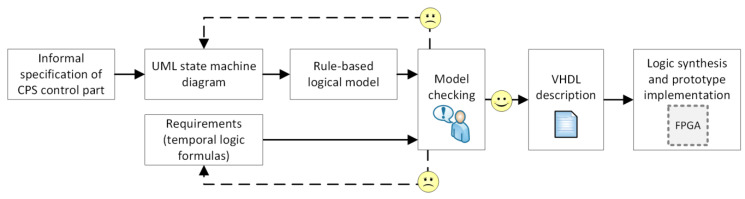
UML state machine diagrams and their verification in the CPS design: schema of the proposed approach.

The proposed approach includes the use of the abstract rule-based logical model. Indeed, the UML state machine diagram is initially written as the rule-based model. Then it is transformed into the verifiable model (in the nuXmv format). The verifiable model is next validated against some user-defined requirements, specified as temporal logic formulas, and preferably written by another design team. If any of the requirements cannot be satisfied, the specification together with the requirements list is revised to check what should be corrected. Although it is often assumed that the unsatisfied requirements indicate some errors in the specification, it can also happen that the requirements that do not hold are not valid and should be corrected (which is illustrated in the case study example). After successful formal verification, the (validated and correct) rule-based logical model is transformed into synthesizable model in VHDL hardware modelling language, ready to be used for prototype implementation in FPGA devices. The schema of the proposed idea is illustrated in Figure 1.

In the article the focus is put on the beginning of the design process, namely, on the construction of a rule-based logical model from the state-based specification of the CPS control part. The further steps of the design, including model checking of a rule-based logical model and its synthesis, are common for the abstract rule-based model. The transformation of rule-based logical model into a verifiable model and a synthesizable model is shortly described in Section 3.4 and illustrated in Section 3.5.

### 3.3. Rule-Based Representation of a State-Based Control Part of a CPS

**Figure 2:**
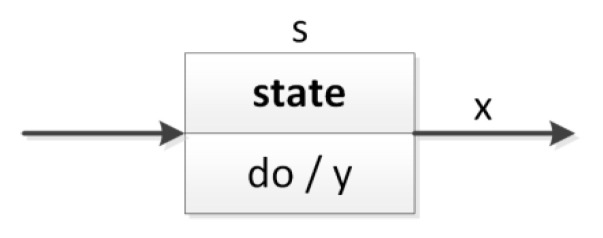
A state in the UML state machine diagram with input and output signals.

The control part of a cyber-physical system specified as a UML state machine diagram is easy to understand, and at the same time, it includes essential information regarding the technical implementation, such as input and output signals. Input signals may come from the environment itself (e.g., sensors), from the user (e.g., operator console) or from other parts of the CPS (e.g., computer vision system). Output signals, in turn, control various objects that are present in the real physical world (e.g., motors, valves, alarm sounds). A UML state machine consists of some states connected with each other, where transitions between them may be carried out under some defined circumstances. A sample state (s) is shown in Figure 2, where during the activity of that state the *y* output signal is active, and the transition to another state of the system is made when the *x* input signal is active (a guarded transition).

The first step in designing the control part of a CPS is taken with the writing of the state-based specification, here using the UML (version 2.x). Then, according to the proposed method, the UML state machine diagram is written as an abstract rule-based logical model, which is done according to Algorithms 1–5. The logical model consists of five sections, each one related to another aspect. Let us describe the particular sections of the rule-based representation and how to construct them.

#### 3.3.1. Definition of Variables

The abstract rule-based logical model starts with the definition of used variables, namely, states of the UML state machine diagram and all input and output signals occurring in the control part of the CPS. Hence, in this step only the structure of the state machine diagram is needed, and in particular, the list of its states, input signals (assigned to transitions) and output signals (assigned to states). The first section of rule-based representation is constructed according to Algorithm 1.
**Algorithm 1.** Generation of the *VARIABLES* section in the rule-based logical model.**Require:** Structure of the UML state machine diagram **Ensure:** Construction of the *VARIABLES* section in the rule-based logical model1:**add keyword*** VARIABLES*2:**add keyword** *places:*              // *definition of states (as places)*3:**for all** *s_i_∈ S***do**4:   add *s_i_* to the set;            ⇨ *places: s_0_, s_1_, s_2_, …*5:**end for**6:**add keyword** *inputs:*             // *definition of input signals*7:**for all** 
*x_j_*
*∈ X* 
**do**
8:   add *x_j_* to the set of inputs;      ⇨ *inputs: x_0_, x_1_, x_2_, …*9:**end for**10:**add keyword** *outputs:*             // *definition of output signals*11:**for all** 
*y_k_*
*∈ Y* 
**do**
12:   add *y_k_* to the set of output;       ⇨ *outputs: y_0_, y_1_, y_2_, …*13:**end for**

#### 3.3.2. Initialization of Variables

The second section of the rule-based model is the initialization of used variables. Here, initially only a pseudo-initial state is active (labelled as *s*0); all input and output signals are marked as inactive. Input signals (and in particular their values) coming in from the real world environment are manipulated in the logical model in order to facilitate then the model checking process. The second section of rule-based representation is constructed according to Algorithm 2.
**Algorithm 2.** Generation of the *INITIALLY* section in the rule-based logical model.**Require:** Variables in the rule-based logical model and initial state indication **Ensure:** Construction of the *INITIALLY* section in the rule-based logical model1:**add keyword** 
*INITIALLY*
2:set the initial state by writing *s*_0_        // *set initial pseudo-state active*3:**for all other** *s_i_∈ S* **starting from** *i =* 1 **do**    // *and other inactive*4:   write *!s_i_;*5:** end for**6:**for all** *x_j_*∈ *X* **do**               // *set all input signals initially as inactive*7:   write *!x_j_;*8:**end for**9:**for all** *y_k_*∈ *Y* **do**               // *set all output signals initially as inactive*10:   write *!y_k_;*11:**end for**

#### 3.3.3. Transitions

The third part of the rule-based logical model is the most essential one and is related to transitions occurring in the system. Each transition is labelled in the state machine diagram and is written as a separate rule with the preceding and following states. Here, both the states and the input signals (if present as transition conditions) are taken into account. Rule’s pre-conditions and post-conditions are separated by an arrow and temporal logic operator *X,* with the meaning that if the pre-conditions are met, then (next) the post-conditions will be realized. The third section of the rule-based representation is constructed according to Algorithm 3.
**Algorithm 3.** Generation of the *TRANSITIONS* section in the rule-based logical model.**Require:** Variables in the rule-based logical and transitions in the UML state machine diagram **Ensure:** Construction of the *TRANSITIONS* section in the rule-based logical model1:**add keyword** 
*TRANSITIONS*
2:**for all** *t_m_∈* *T* **do              **// *define one rule for each transition*3:   add label *t_m_:*4:   add the preconditions **         **// *specify preceding states & input signals*5:   add keyword *-> X*6:   add the postconditions **          **// *specify following states*
7:**end for**

#### 3.3.4. Input Signals

The fourth part of the rule-based notation regards the input signals and simulates the activity of them in the preceding states. It should be noted that in general the input signals should not be manipulated; however, in the proposed approach it is done only to facilitate formal verification. It is used only for the model checking process in order to eliminate the state explosion problem. This section is omitted in the generation of a synthesizable model in VHDL. The fourth section of rule-based representation is constructed according to Algorithm 4.
**Algorithm 4.** Generation of the *INPUTS* section in the rule-based logical model.**Require:** UML state machine diagram, focus on guarded transitions **Ensure:** Construction of the *INPUTS* section in the rule-based logical model1:**add keyword** 
*INPUTS*
2:**for all** 
*t_m_*
*∈ T* 
**do**
3:   **if** *t_m_* has assigned a condition **then**
4:     assign input signal to preceding state by writing 5:       *s_i_ -> !input | input;* //
*if state s_i_ is active, then the input signal may be activated or deactivated* **end if**
6:**end for**

#### 3.3.5. Output Signals

The last (fifth) part of the rule-based logical model regards the output signals and defines which of them are active in which state of the system. Hence, here both the states from the UML state machine diagram and the corresponding outputs signals are taken into account. The last section of rule-based representation is constructed according to Algorithm 5.
**Algorithm 5.** Generation of the *OUTPUTS* section in the rule-based logical model.**Require:** States of the UML state machine diagram **Ensure:** Construction of the *OUTPUTS* section in the rule-based logical model1:**add keyword** 
*OUTPUTS*
2:**for all** 
*s_i_*
*∈ S* **do**
3:      **if** *s_i_* has assigned an output signal **then**4:      write *s_i_ -> output;*5:   
**end if**
6:**end for**

### 3.4. General Description of Transformations into a Verifiable Model and a Synthesizable Model

Let us first give a short introduction to how verifiable and synthesizable models are generated. They both are based on the rule-based logical model and are generated fully automatically with the application of the developed *m2vs* tool (*model to verification and synthesis*), which allows the elimination of errors related to hand-written coding. Using strictly defined rules of transformations ensures both that the obtained models are consistent with each other and that the prototype implementation reflects the formally verified specification.

**Figure 3:**
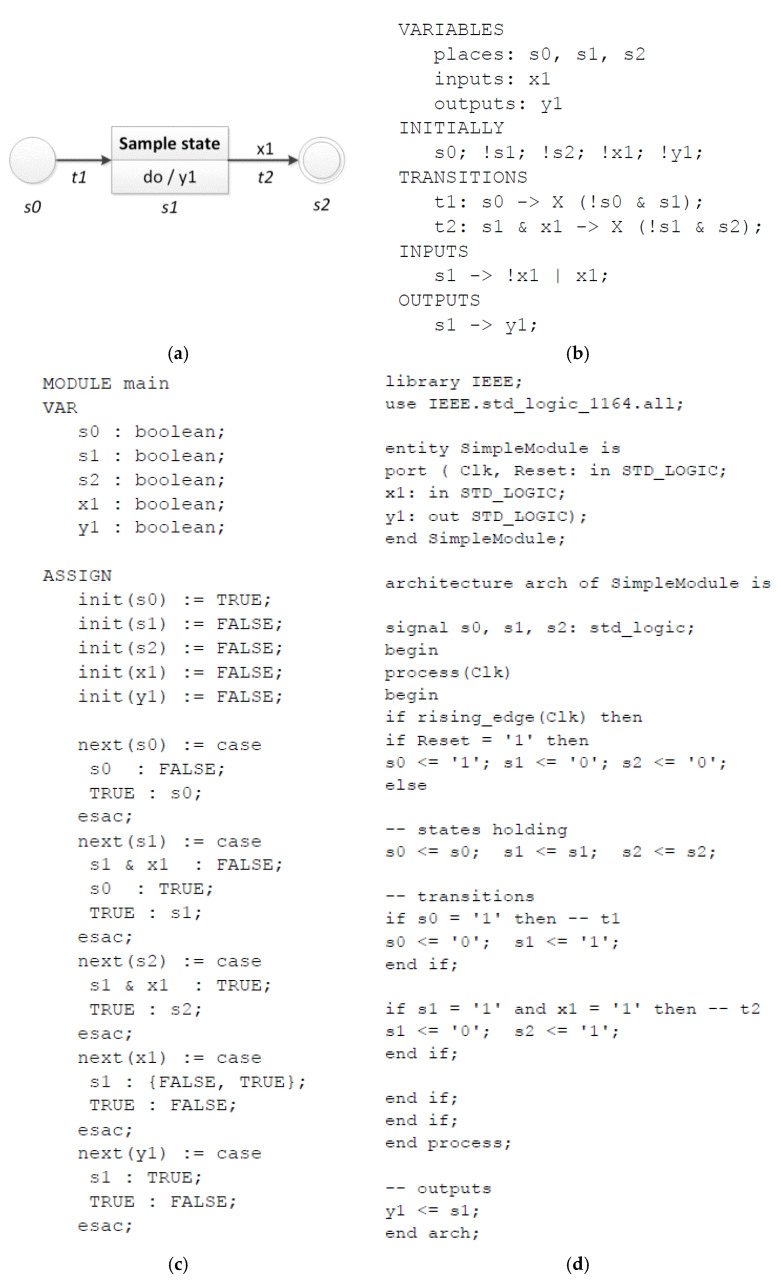
Illustration of the algorithms: a simple state machine diagram (**a**), the corresponding rule-based logical model (**b**), a generated verifiable model in the nuXmv format (**c**) and a generated synthesizable model in VHDL (**d**).

A rule-based logical model is transformed into a verifiable model sequentially, reading line by line of the text description. A verifiable model in the nuXmv format involves two main sections, namely, variables definition and assignments. Since its structure is quite similar to the structure of the rule-based model, each section of the logical model is reflected in the appropriate section of the nuXmv model. All variables (section *VARIABLES*) are defined as separate Boolean variables. All assignments of initial values (section *INITIALLY*) are done at the beginning of the *ASSIGN* section of the nuXmv model. Then, for all variables a transition relation statement is created. Variables related to states (section *TRANSITIONS*) which occur in a rule’s pre-condition become inactive (value *false*), while the ones that occur in a rule’s post-condition become active (value *true*). Variables related to input signals (section *INPUTS*) are supposed to change their values only in appropriate states (in order to eliminate the state explosion problem in model checking). Finally, variables related to output signals (section *OUTPUTS*) are assigned the *true* value in corresponding states. Please see Figure 3c for an automatically generated verifiable model of a simple rule-based logical model in the nuXmv format.

The transformation of a rule-based model into a synthesizable model is also realized sequentially, reading line by line of the text description. Appropriate keywords of the VHDL language are inserted where necessary. Input ports of clock and reset signal are defined, followed by the definition of all input and output signals (from section *VARIABLES*). States variables are defined as internal signals. Then, the initial assignments of state values (section *INITIALLY*) are written. For all rules (section *TRANSITIONS*) an “if” statement is created. Section *INPUTS* is not reflected in the VHDL code, since input signals may not be manipulated. Finally, all output rules (section *OUTPUTS*) activate an output signal by appropriate active states. Please see Figure 3d for an automatically generated synthesizable model of a simple rule-based logical model in the VHDL.

For detailed descriptions of transformation algorithms please refer to [12].

### 3.5. Illustration

The proposed algorithms are briefly illustrated with an example of a simple state machine diagram consisting of one state, one input and one output signal, as shown in Figure 3. The labelled state machine diagram (a) results in the following rule-based logical model (b), which is then automatically transformed into a verifiable model (c) and a synthesizable model (d) with the developed *m2vs* tool.

All parts of the rule-based logical model are reflected in the automatically generated verifiable model, with the fourth part of it (with input signals) being used only to eliminate the state explosion problem. To automatically generate the synthesizable model in VHDL language, all parts except the fourth one are used, and additionally the initial values of the input signals from the second part are not taken into account.

It should be noticed that the rule-based logical model is much shorter than the resulting verifiable and synthesizable models. Therefore, it is less error-prone and easier to use. Since model transformations are done automatically, errors related to hand-written coding of nuXmv model or VHDL can also be eliminated.

## 4. Case Study of a Modern Manufacturing Automation System

**Figure 4:**
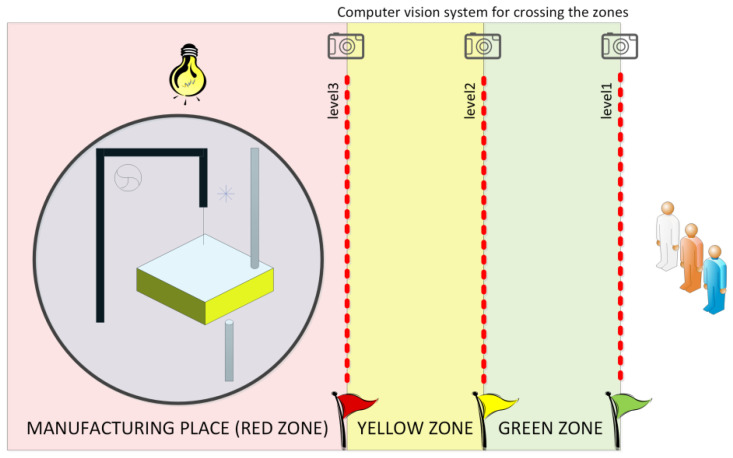
A case study example of a manufacturing cyber-physical system.

Let us illustrate the presented approach with a case study example of a modern manufacturing automation system shown in Figure 4 (originally introduced in [73]). The production plant includes a milling machine for cutting out wooden shapes (described in detail in [74]), which is situated in the manufacturing location (red zone). The cyber-physical system involves also a computer vision system, which is responsible for monitoring of crossing the boundary lines between the safety zones. Some safety rules are set for each group of employees and appropriate safety mechanisms are then activated. Everyone may stay in the green zone, while ordinary users and shift supervisors may enter the yellow zone (with different consequences), but only supervisor service employees or managers may enter the red zone. In the last case the safety mode is launched with slowing down the production and turning on an additional lightning system. The control part of the manufacturing cyber-physical system is connected with both the production and the computer vision system.

### 4.1. Specification with UML State Machines

**Figure 5:**
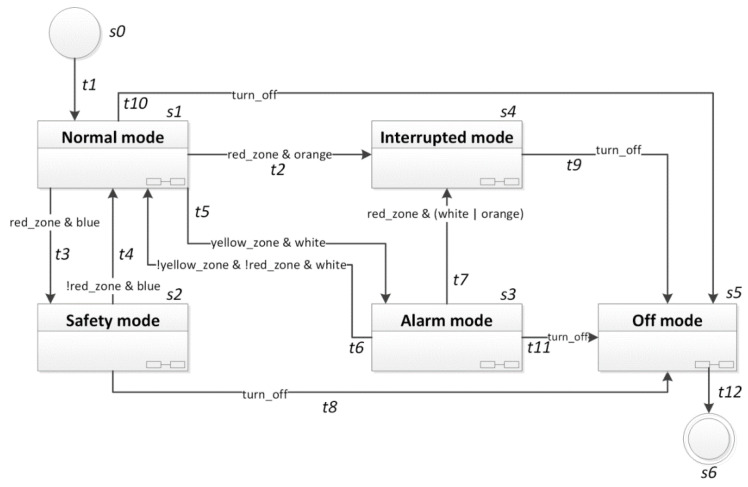
A high-level UML state machine diagram for the case study.

The briefly described sample manufacturing cyber-physical system can be specified with the use of UML state machine diagrams (in version 2.x). The corresponding high-level state machine diagram is shown in Figure 5.

It includes five composite states with hidden decomposition (*s*1*–s*5), where each of them is another working mode and can be specified in detail as a separate specification (in the form of a state machine diagram, or thanks to the rule-based logical model, also in the form of a Petri net). The changes between working modes occur in response to some events generated by the computer vision system monitoring crossing of the lines between different safety zones. It should be noted that returning from a deeper zone to a previous one is always recognized when no more unprivileged employees remain in that zone. Moreover, transitions to more dangerous zones always have higher priority (for the sake of readability the priorities are not explicitly written on the diagram).

**Figure 6:**
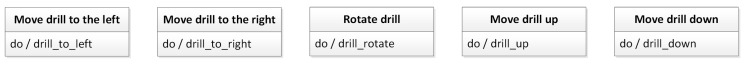
Several simple states within the “Normal mode”.

In the UML state machine diagram, input signals coming from the computer vision system are conditions for transitions between the composite states. Inside each composite state, some simple states can be defined, and other input signals can occur, together with output signals controlling the executive elements. Figure 6 presents several simple states within the “Normal mode” state with particular output signals.

### 4.2. Construction of the Rule-Based Logical Model

**Figure 7:**
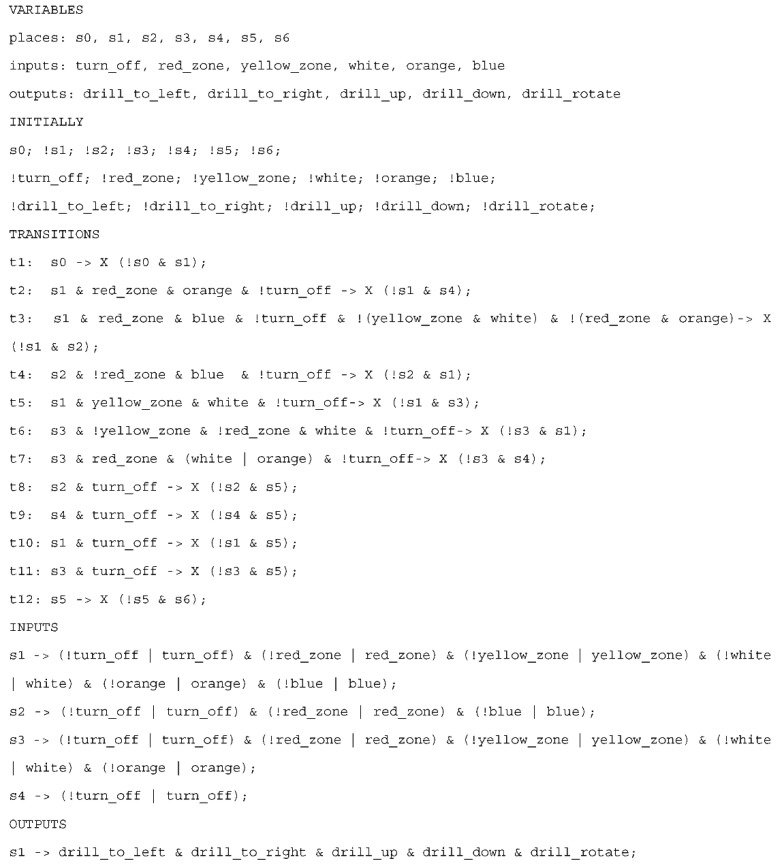
Rule-based logical model for the case study, with partial possible output signals.

The labelled state machine diagram (here in the form of a high-level diagram) is written as the rule-based logical model, as shown in Figure 7. Thus, each state and input signal (from Figure 5) are defined and initialized. Moreover, the sample output signals that are active at any time within the “Normal mode” composite state *s*1 (Figure 6) are here also defined and initialized. Note that when considering the high-level diagram, all output signals within a composite state are set active with that state, which means that inside that state some of them may be active at any time (i.e., it is always possible that the output signals will be active within that composite state) and the mutual correlation between them is not determined (i.e., it does not mean that all output signals are active simultaneously). The rule-based logical model for the UML state machine diagram of the case study (with prioritized transitions and sample simple states inside one composite state) consists of 28 lines.

In the first section all the states *s*0, …, *s*6 are defined, and used input and output signals. Then, the variables are initialized. If a variable name is preceded by an exclamation mark, it means that initially that variable value is set to 0 (Boolean value *false*), otherwise it is set to 1 (Boolean value *true*). Thus, the initial state of the system is *s*0 and all input and output signals are inactive. Then, all the transitions from a UML state machine diagram are described as rules; here we have twelve rules covering the twelve arcs in the diagram. In each rule there is a pre-condition and a post-condition. The pre-condition may involve only the active state (see, e.g., rule *t*1) or an active state together with transition conditions (see, e.g., rule *t*2). The post-conditions (after the *next* temporal logic operator) define how the system state changes, in particular which states become inactive (state name preceded by an exclamation mark) and which states become active (state name without an exclamation mark). For example, rule *t*11 specifies that whenever state *s*3 (alarm mode) is active and the turn-off signal is received (transition condition), then state *s*3 (alarm mode) becomes inactive and state *s* (off mode) becomes active.

The next two sections in the rule-based model regard input and output signals. Please note that the changes of input signals are used only in formal verification (in order to eliminate the state explosion problem), they are not considered at all in a synthesizable model. The changes of input signal values are expected in the states preceding the particular transitions; e.g., since it is assumed that the interrupted mode (state *s*4) may change only to the off mode (state *s*5), when state *s*4 is active, only the turn off signal is considered (changes of other input signals do not influence the system state, and hence, they are not taken into account). Finally, the last section declares which output signals are active within each state, thus in the example of the simplified-use case there are only output signals referring to the normal mode (state *s*1).

The abstract rule-based logical model of the control part (with 28 lines of code) is then automatically transformed into a verifiable model in the nuXmv format with 127 lines of code (approx. 4.5 times longer). The generation of a verifiable model is achieved with the developed *m2vs* software, which has been used for the experiments. After generation of a verifiable model it is necessary to prepare the extensive list of user-defined requirements.

### 4.3. Formal Verification of User-Defined Requirements

**Figure 8:**
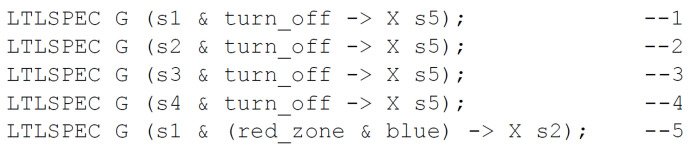
Sample requirements for formal verification of the case study.

To perform model checking of the specification (with the nuXmv tool), the already available verifiable model needs to be extended with a requirements list. The requirements are defined as temporal logic formulas (here, the LTL) and include first of all the safety and liveness properties, i.e., situations that cannot happen and the ones that will eventually happen. Due to the high-level specification with composite states, the defined properties focus mostly on the influence of input signals on the transitions between states, and with the correlation between input signals and activity of particular output signals. The correlation between output signals within the same composite state is not considered and can be verified using the detailed (decomposed) specification of that state and its rule-based logical representation. Sample requirements for the CPS case study are presented in Figure 8. They are specified in linear temporal logic with the global operator *G*, with the meaning that these properties must hold globally. Properties 1–4 check whether priorities of transitions are correctly set—that is, whether an active turning-off signal always results in changing system state into the off mode (state *s*5). Model checking of these requirements for turning off the production reveals that they are all satisfied. The last property says that a blue uniform in the red zone should result in a system state change into the safety production mode (state *s*2). This property does not hold and the undesired situation must now be analyzed in detail.

**Figure 9:**
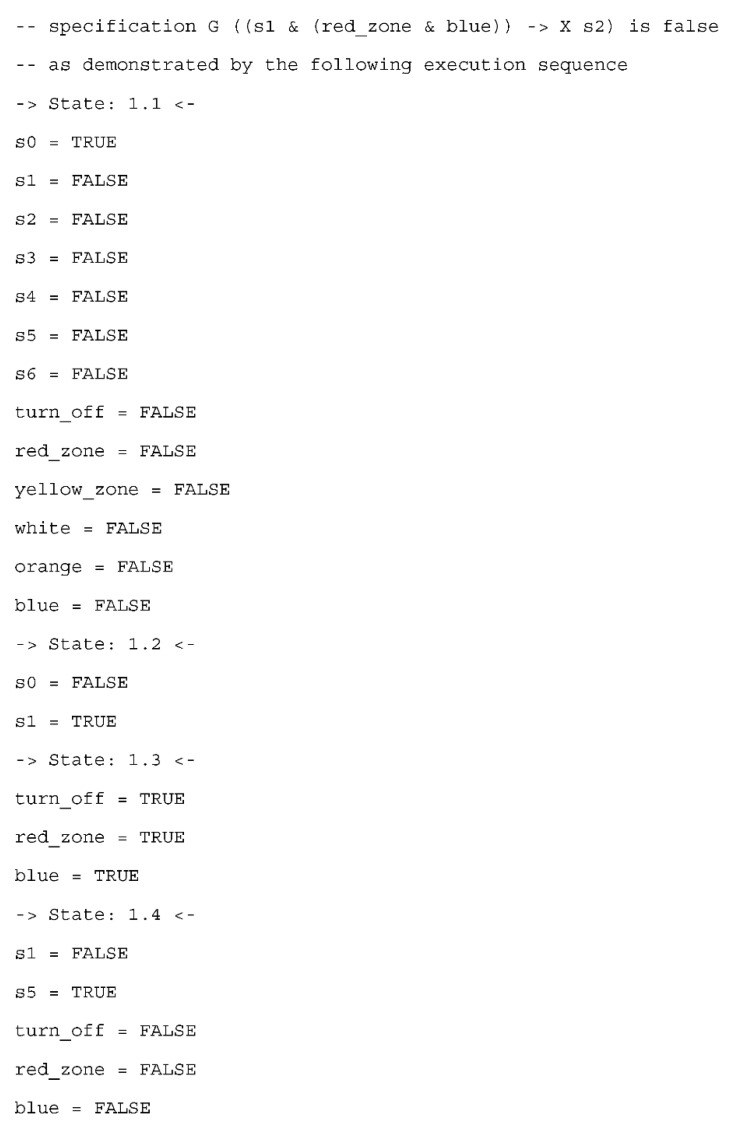
Generated counterexample for a normal mode with blue uniform in the red zone leading to safety mode and turning off signal leading to off mode.

Let us consider the benefits of model checking for such a high-level state-based specification with the last property, which is not satisfied. As a result, a counterexample is generated with a trace leading to the undesired situation (Figure 9, without output signals). The analysis of this reveals that in the normal mode (state 1.2 in the counterexample), despite the active signal of a blue uniform in the red zone (state 1.3 in the counterexample), the safety mode is not always launched. Indeed, it can happen that turning off the production is activated at the same time (state 1.3 in the counterexample), and as a result the normal mode changes directly into the off mode (state 1.4 in the counterexample). This is obviously proper behavior of the system (as transitions into the off mode are the most privileged), but it shows that much attention should be paid both to the specification of the control part and its requirements. These two factors are needed for formal verification and are often delivered by other development teams, thus different points of view are compared with each other and should be consistent. Hence, in the considered situation, the requirement for entering the red zone by a blue uniform has to be revised.

**Figure 10:**
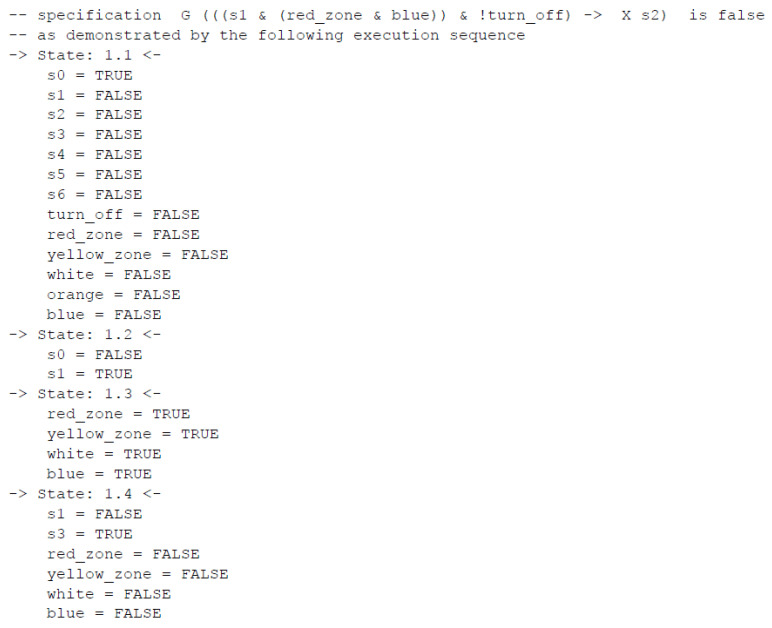
Generated counterexamples for changing into the safety production mode with a white uniform in the yellow zone leading to alarm mode.

Then, the appropriate condition of turning off is added (the turning-off signal is inactive since it has a higher priority in the system), and the requirement is corrected, stating now that a blue uniform in the red zone and without turning-off signal should result in a system state change into the safety production mode (state *s*2). However, model checking reveals that this is still not satisfactory (Figure 10, without output signals), as it can happen that at the same time that a white uniform enters a yellow zone (state 1.3 in the counterexample), and as a result the normal mode changes directly into the alarm mode (as a mode with the higher priority, state 1.4 in the counterexample). Hence, the requirement has to be revised again.

Look further: clearly it is also possible that an orange uniform enters a red zone causing interrupted mode (state *s*4, with higher priority) to be activated.

Therefore, to ensure proper system behavior for activation of the safety mode, the correct requirement should exclude higher priority transitions and should be finally written as:

LTLSPEC G (s1 & (red_zone & blue) & !(yellow_zone & white) & !(red_zone & orange) & !turn_off -> X s2);

This clearly shows that in the model checking process only the defined requirements are checked, and the specification of them is as important as the state-based specification of the control process.

### 4.4. Further Steps

After successful model checking, the development process of the control module in the cyber-physical system may proceed and the rule-based logical model can be transformed automatically into the synthesizable model in the VHDL language; then simulated; and finally, implemented.

The abstract rule-based logical model of the control part, constructed in Section 4.2, is automatically transformed into a synthesizable model in the VHDL language ready to be implemented in the FPGA device with 82 lines of code (approx. three times longer). The generation of a synthesizable model for prototype implementation is achieved with the developed *m2vs* software. Please note that a prototype implementation was obtained; therefore, any performance or optimization issues were not considered.

**Figure 11:**
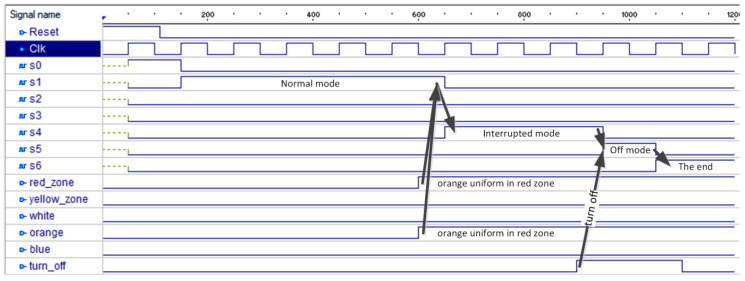
Simulation results of the case study.

The resulting program in the VHDL language was simulated in the Active-HDL environment to confirm the correct hardware description. Sample simulation results for changing the normal operation mode (state *s*1) into the interrupted mode (state *s*4), then into the off mode (state *s*5) and finally reaching the terminal state (state *s*6), are shown in Figure 11. The changes in the CPS occur when an orange uniform is detected in the red zone and then when turning off is activated (which is indicated by appropriate input signals). The terminal state is reached automatically from the off mode.

Finally, the VHDL code was implemented using *Spartan 3E Starter Board* (with emulation of real sensors and actuators). It should be noted that any performance issues are not considered here and an additional optimization of occupied logic blocks can be performed. Some other aspects of the implementation can also be investigated further, e.g., regarding timing or security; they are, however, out of scope of this paper.

## 5. Discussion

Classical manufacturing systems nowadays are enriched with the capabilities of the cyber-physical systems, in which information from all related perspectives is closely monitored and synchronized between the physical factory and the cyber part, and have evolved into Industry 4.0 [9]. The integration of cyber and physical parts plays an important role in state-of-the-art manufacturing systems [75]. Examples of some practical applications of the 4.0 industry are very diverse, and include steel [76] and mining [77] companies, smart jacket [78] and sunroof ambient light [79] production systems and construction industry applications [80]. The proposed formal verification of a CPS control module using an abstract rule-based logical model fits, then, perfectly into the contemporary trends and may be used in modern manufacturing automation systems.

What should be emphasized is the fact that the proposed approach is suited for general cyber-physical systems. Although the specifics of CPSs are limited to input and output signals and to interaction with the environment, it has been shown that the proposed design methodology may be part of a complex project of CPS development. The interaction between all parts of a cyber-physical system is a key issue that must be precisely defined and investigated in order for the whole system to function correctly. Here, we show that the control part communicating with the other parts using input and output signals may be specified in a simple user-friendly way with UML state machine diagrams, but at the same time it can be formally verified using model checking. Hence, the quality of a CPS control scheme is increased and as is the assurance that all requirements that have been defined are fulfilled and the specification goals met.

The formalization of UML diagrams has also been considered earlier in the literature. However, the older existing approaches concern statecharts (for example [81]) that are said to be a precursor of state machine diagrams, but there exist some important differences between the two types of diagrams. The newer ones deal with the state machine diagrams; however, they focus mostly either on software or take into account just the verification. For example, in [82] formal semantics of a UML state diagram and automatic verification based on a Kripke structure is proposed for software architecture. Other papers discuss UML diagrams, along with their SYSML/MARTE profiles, for requirement specifications (e.g., model-based property specifications for early design verification [83]) or temporal property verification (e.g., for critical real-time systems [84]).

What distinguishes the proposed approach to UML state diagram formalization in the form of rule-based logical model from the other ones is: firstly—the support for the current standard of Unified Modelling Language; secondly—the support for formal verification with model checking; thirdly—the support for synthesis and implementation; and lastly—the support for modular models (using composite states). All the aforementioned benefits are applicable to the design of the control scheme of a cyber-physical system.

It should be noted that the proposed rule-based specification of the control scheme can be automatically transformed into a verifiable model and a synthesizable model (where both of them are consistent with each other); hence, the development time is reduced and the amount of hand-made errors is significantly limited. Additionally, the same rule-based logical model can be used also for other types of control scheme specification, namely, Petri nets, which has already been shown in [12], and therefore, if needed, two different forms of specification may be used in one project without any problems.

The proposed approach is not limited to state-based control modules of cyber-physical systems. It may be generalizable to other control systems, not only in the domain of CPS, with binary input and output signals. The rule-based logical model is abstract, so it may reflect also other forms of control process specification, such as, control of interpreted Petri nets.

On the other hand, the proposed solution also has several limitations. First of all, the proposed prototyping flow relies only on FPGA devices; hence, it is platform-dependent. Fortunately, no knowledge of VHDL hardware description language is needed, as the fully synthesizable code is generated automatically, based on the logical model, and does not need to be dealt with manually. Moreover, the presented approach is based on the UML language; hence, some specialized knowledge from the designer is required. However, taking into account the popularity of UML in the industry, the proposed method can still be used in practice by a large number of engineers.

## 6. Conclusions

In the paper, the novel rule-based approach for formal verification of a control scheme for a cyber-physical system has been proposed. The main idea is based on the use of an abstract logical model, which can be automatically transformed into a verifiable model, and at the same time also into a synthesizable model. The control scheme is presented as a state-based user-friendly specification and is sketched as a UML state machine diagram, which is easy to use, to interpret and to understand. The state-based specification is then written as the rule-based logical model, which can be done at different levels of generality (as is the case with the UML diagrams)—starting from composite states and ending with detailed simple states. Various perspectives offer a wide range of options for checking a variety of scenarios and for focusing on particular elements of a control system (e.g., correlation between output signals or transitions in the systems occurring after some events). The contribution was illustrated with a case study of a modern manufacturing automation system.

The highlights of the paper can be shortly summarized as follows: (a) a UML state-based specification of the control scheme of a CPS is given, (b) the detailed algorithms of rule-based model generation are provided, (c) the method of how to establish an abstract rule-based logical model is explained, (d) the specification is formally verified and then a prototype implementation in an FPGA device can be performed and (e) the case study illustrates the proposed method.

Thanks to the abstract rule-based logical model, the control scheme of a cyber-physical system can be prepared using not only one specification form, which is a great advantage. Indeed, it is possible to combine in one design project both UML state machine diagrams and Petri nets (used at various generality levels), with both of them being able to be written as rule-based models. Additionally, these rule-based specifications can then be formally verified against user-defined requirements and quickly implemented in FPGA devices.

The article clearly shows that much attention should also be paid to the definition of requirements. In the model checking process, the system model is compared with the list of requirements, and if any of the latter is not satisfied, then the requirements and the system model should be revised and appropriate corrections should be made.

Various practical experiments have been conducted, focusing especially on the formal verification of the state-based control scheme of cyber-physical systems. The control process specifications were delivered in the form of UML state machine diagrams. The sample specifications were formally verified using the abstract rule-based logical model and the model checking technique. The resulting programs in the VHDL language were simulated. The tests showed that the proposed approach can be used in practice for both the formal verification of control scheme of a CPS and for automatic prototype implementation in an FPGA device. The tests have also confirmed that it is possible to use in one design different forms of specification, i.e., UML state machine diagrams and Petri nets, since the abstract rule-based logical model is common to them both.
